# Severe malaria - a case of fatal *Plasmodium knowlesi *infection with *post-mortem *findings: a case report

**DOI:** 10.1186/1475-2875-9-10

**Published:** 2010-01-11

**Authors:** Janet Cox-Singh, Jessie Hiu, Sebastian B Lucas, Paul C Divis, Mohammad Zulkarnaen, Patricia Chandran, Kum T Wong, Patricia Adem, Sherif R Zaki, Balbir Singh, Sanjeev Krishna

**Affiliations:** 1 Division of Cellular and Molecular Medicine, Centre For Infection, St George's University of London, Cranmer Terrace, London SW17 0RE, UK; 2 Malaria Research Centre, University Malaysia Sarawak, Kuching, Sarawak, Malaysia; 3 Department of Forensic, Queen Elizabeth Hospital, Kota Kinabalu, Sabah, Malaysia; 4 Department of Histopathology, KCL School of Medicine, St Thomas' Hospital, London, UK; 5 Department of Pathology, Faculty of Medicine, University of Malaya, Kuala Lumpur, Malaysia; 6 Infectious Diseases Pathology Branch, Centers for Disease Control and Prevention, Atlanta, Georgia, USA

## Abstract

**Background:**

Zoonotic malaria caused by *Plasmodium knowlesi *is an important, but newly recognized, human pathogen. For the first time, post-mortem findings from a fatal case of knowlesi malaria are reported here.

**Case presentation:**

A formerly healthy 40 year-old male became symptomatic 10 days after spending time in the jungle of North Borneo. Four days later, he presented to hospital in a state of collapse and died within two hours. He was hyponatraemic and had elevated blood urea, potassium, lactate dehydrogenase and amino transferase values; he was also thrombocytopenic and eosinophilic. Dengue haemorrhagic shock was suspected and a post-mortem examination performed. Investigations for dengue virus were negative. Blood for malaria parasites indicated hyperparasitaemia and single species *P. knowlesi *infection was confirmed by nested-PCR. Macroscopic pathology of the brain and endocardium showed multiple petechial haemorrhages, the liver and spleen were enlarged and lungs had features consistent with ARDS. Microscopic pathology showed sequestration of pigmented parasitized red blood cells in the vessels of the cerebrum, cerebellum, heart and kidney without evidence of chronic inflammatory reaction in the brain or any other organ examined. Brain sections were negative for intracellular adhesion molecule-1. The spleen and liver had abundant pigment containing macrophages and parasitized red blood cells. The kidney had evidence of acute tubular necrosis and endothelial cells in heart sections were prominent.

**Conclusions:**

The overall picture in this case was one of systemic malaria infection that fit the WHO classification for severe malaria. Post-mortem findings in this case were unexpectedly similar to those that define fatal falciparum malaria, including cerebral pathology. There were important differences including the absence of coma despite petechial haemorrhages and parasite sequestration in the brain. These results suggest that further study of knowlesi malaria will aid the interpretation of, often conflicting, information on malaria pathophysiology in humans.

## Background

The underlying pathophysiology of malaria is not well understood despite considerable and prolonged international research effort. This effort is largely driven by the need to reduce the impact of *Plasmodium falciparum *on human life, particularly in African children [1,2]. Part of the difficulty in studying the pathophysiology of *P. falciparum *malaria is the lack of a permissive animal model and a comparator disease in man. With the recent discovery of severe malaria, caused by the zoonotic parasite *Plasmodium knowlesi *in the human population of Southeast Asia [3,4], it is now possible to link findings in primates and humans through this common agent.

In the natural primate hosts (long and pig-tailed macaques), *P. knowlesi *causes asymptomatic low-grade parasitism. In contrast, hyperparasitaemia and death ensue in the well-used Rhesus experimental model [5]. Consequently, there is much information on experimental *P. knowlesi *malaria, including organ pathology in various animal models [6-8]. Natural *P. knowlesi *infections of humans were formerly missed because of morphological similarity with *Plasmodium malariae *[9,10]. Now clinical descriptions of knowlesi malaria encompass a spectrum of disease ranging from uncomplicated to fatal malaria [3,4].

The pathophysiology of severe knowlesi malaria in humans is undescribed, but nonetheless important for several reasons. First, to provide improved guidelines for the diagnosis, treatment and management of severe knowlesi cases. Secondly, when severe knowlesi infection is compared with severe falciparum malaria, this can help clarify determinants of severe disease. Thirdly, it is possible that we are observing a *P. knowlesi *vertebrate host switch from Southeast Asian macaques to a full-blown emergence into the human population [11,12]. Therefore, it is important to understand properly the disease caused by *P. knowlesi *to assist strategies to reduce health-impacts if human-to-human transmission of this virulent pathogen is established.

The clinical, laboratory and, for the first time, post-mortem findings in a fatal case of knowlesi malaria are reported here. The histopathology in various organs is explained within the context of *P. knowlesi *biology and comparisons with the salient features made with what is known of the pathophysiology of severe falciparum malaria [13-16].

## Case presentation

### History and examination

A forty year-old male was brought to the Queen Elizabeth Hospital, Kota Kinabalu, Sabah (8.30 am) in a state of collapse. He was unable to give a history himself or to stand. On examination his blood pressure was unrecordable and oxygen saturations were recorded as low.

The patient had no past medical history. He had spent two weeks in the jungles of Borneo before leaving for an urban setting. Ten days after leaving the jungle he experienced the onset of fever and body aches. Two days later, he sought treatment at a government outpatient clinic continuing to complain of fever and myalgia. A specific diagnosis was not made and he was able to work although he developed rashes the next day. He remained unwell for the next two days, when he presented in a state of collapse and had developed abdominal pain.

Resuscitation measures were begun and the patient was immediately intubated, given adrenaline/atropine and sodium bicarbonate. On examination and after resuscitation measures his vital signs were: symmetrical air entry into lungs, BP 58/44 mm Hg, pulse rate 40-50 per minute with poor peripheral perfusion and cyanosis. Heart sounds were normal. He had generalized petechiae and his abdomen was tense and distended.

Resuscitation measures continued for one hour, during which time "coffee grounds" were observed in the nasogastric aspirate. The patient became asystolic after one hour and although cardiopulmonary resuscitation was given for a further 20 minutes, there was no response. The patient was pronounced dead two hours after admission.

Dengue haemorrhagic shock was suspected and a post-mortem examination was performed approximately 24 hours later.

### Laboratory findings

**Figure 1:**
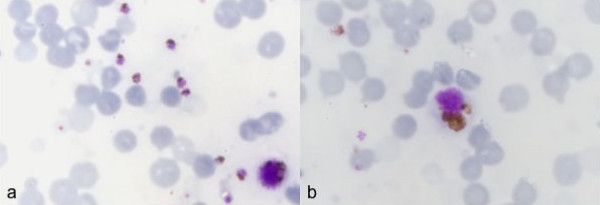
**Thin blood film showing mostly late trophozoites of *P. knowlesi *in poorly defined erythrocytes (1a) and heavily pigmented monocyte (1b)**. Note that the blood film was prepared 24 hours post mortem and shows red cell ghosts, particularly among parasite infected red blood cells.

Laboratory results are summarized in Table 1. The patient was not anemic, but was thrombocytopenic and had an eosinophilia. He was also hyponatraemic and had elevated blood urea, potassium, lactate dehydrogenase and amino transferase values. Serum creatinine was not available. A blood sample taken 24 hours post-mortem showed >10% of erythrocytes infected with predominantly pigmented parasites (Figure 1a). Heavily pigmented monocytes were also present (Figure 1b). *Plasmodium knowlesi*, as a single species infection, was confirmed by nested-PCR [9]. Post-mortem dengue serology was negative (Table 1) and dengue, respiratory syncytial virus and enterovirus were not isolated in organ samples.

**Table 1:** Admission laboratory findings

Parameter	Values normal [range]	Values observed
Haemoglobin (g/dL)	[13·5-17·5]	12.7
Platelets (/uL)	[150,000-450,000]	42,800
Haematocrit (%)	[42-54]	36.7
Total white blood cells (/uL)	[4,500-11,000]	10.1
Neutrophils (%)		15.1
Lymphocytes (%)		36.7
Monocytes (%)		12.1
Eosinophils (%)		33.9
Basophils (%)		2.17
Red blood cells (× 10^6^/uL)	[4-5]	4.86
Mean Cell Volume (fL)	[80-100]	75.5
Mean Cell Haemoglobin (pg)	[29-31]	26.1
Mean Corpuscular Haemoglobin concentration (g/dL)	[32-36]	34.6
Mean Platelet volume (fL)	[7.5-11.5]	11.5
Serum sodium (mmol/L)	[135-150]	126
Serum potassium (mmol/L)	[3.3-5.1]	6.9
Serum urea (mmol/L)	[1.0-8.3]	34.1
Serum aspartate amino transferase (U/L)	[<38]	131
Serum creatine kinase (U/L)	[<170]	156
Serum lactate dehydrogenase (U/L)	[240-480]	2777

### Post-mortem examination

External examination showed a well-nourished adult male. The conjunctivae showed tinges of jaundice and the right eye had subconjunctival haemorrhages. There were multiple petechial haemorrhages on the body and venepuncture sites were associated with marked bruising. Coffee ground material was noted in the mouth. Internal examination revealed no tissue oedema or excess fluid in the body cavities.

### Macroscopic pathology

**Figure 2:**
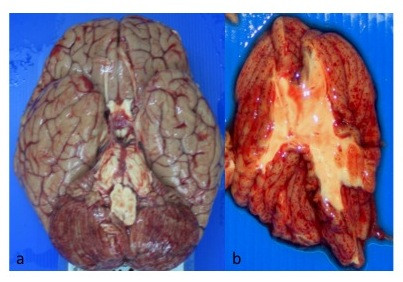
**Gross appearance of the brain**. The outer surface appears dusky with petechial haemorrhages seen on the outer surface of the cerebellum (2a). Cut section of the cerebellum with multiple petechial haemorrhages seen within the cerebellar grey matter (2b).

The external surfaces of the cerebrum were dusky. The cut sections showed multiple petechial haemorrhages. The cerebellum also showed petechial haemorrhages externally and on multiple cut sections (Figure 2a and 2b). The brain stem and upper spinal chord were grossly normal. Both lungs were heavy (weighing on the right 720 g and left 690 g) and cut sections were congested and 'beefy' in appearance. Petechial haemorrhages were present on the endocardium with extensive subendocardial haemorrhages involving the left ventricular wall. The haemorrhages were most prominent at the apex of the heart. The liver (2640 g) and spleen (340 g) were markedly enlarged. The cut surfaces of the spleen were soft and friable. The gallbladder, pancreas and kidneys were grossly normal.

### Microscopic pathology

**Figure 3:**
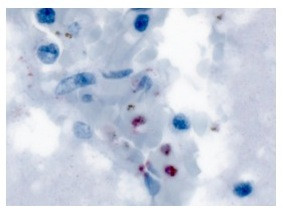
**Plasmodium-specific anti-aldolase immunohistochemistry stained sections from the brain**. Parasites appear red.

Haematoxylin and eosin stained sections from various organs were available for examination. Parasitized red blood cells (PRBC) were abundant although parasite bodies were obscured by haemozoin (malaria) pigment. Chemical removal of pigment and oil immersion (×1,000) magnification revealed trophozoites that were discernibly bigger than those of *P. falciparum*. Immunohistochemistry stained sections from the brain were Plasmodium anti-aldolase positive (Figure 3) and negative for *P. falciparum*- specific staining [17].

**Figure 4:**
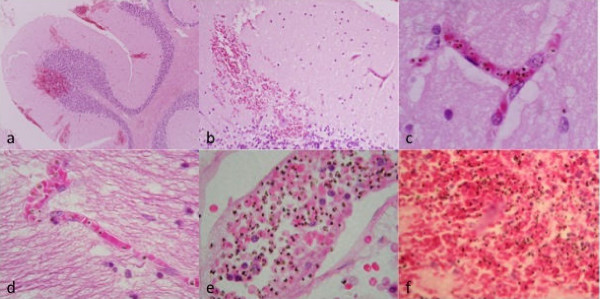
**Haematoxylin and eosin stained sections of the cerebellum (a, b, c, e, f) and cerebrum (d)**. Low power view showing haemorrhages in the grey and white matter × 20 (4a). Haemorrhage in the cerebellar molecular layer × 40 (4b). 4c × 400 and 4d both × 200 show capillaries with sequestered parasitized red blood cells. There was no perivascular inflammation nor intravascular thrombosis. Venule with parasitized red blood cells × 200 (4e) and haemorrhage showing mixture of parasitized and non-parasitized red blood cells × 400 (4f).

Many petechial haemorrhages (up to 600 μm diameter) arising from the rupture of the small vessels of the cerebrum and cerebellum were observed (Figures 4a and 4b) Sequestration of PRBC was evident within small blood vessels (Figures 4c and 4d). Congested larger vessels and areas of haemorrhage showed considerable amounts of malaria pigment (Figures 4e and 4f). Clumps of platelets or evidence of thrombi in vessels were not seen. There was no evidence of vasculitis or perivascular chronic inflammatory reaction in the brain or any other organ examined (heart, kidney, liver, adrenal gland and spleen). There was no evidence of perivascular or diffuse parenchymal oedema in the brain. Diffuse astrocytosis or microgliosis was not observed, nor was there evidence for acute gliotic reactions about the haemorrhages. There was no aggregation of polymorphs in the vessels, no perivascular inflammation, nor generalized encephalitis. There was no diffuse thrombotic microangiopathy, but within one haemorrhage there was probably some fibrin at the site of the vessel. Immunohistochemistry of sections from the brain was negative for CD54 (which stains intercellular adhesion molecule-1, ICAM-1).

**Figure 5:**
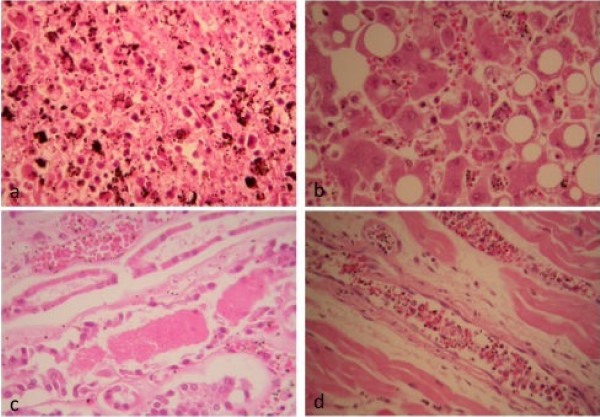
**Haematoxylin and eosin stained extra cerebral tissues**. (5a) spleen × 400 showing red pulp macrophages containing much haemozoin pigment as well as phagocytozed red blood cells. (5b) liver × 400 showing macrovesicular steatosis of hepatocytes without parenchymal inflammation. The sinusoids have abundant parasitized red blood cells as well as Kupffer cells containing haemozoin. (5c) kidney × 200 showing acute tubular necrosis and intratubular casts. Intravascular parasitized red blood cells also visible. The myofibres of the left ventricle of the heart are normal (5d × 200). Small veins and capillaries contain abundant parasitized red blood cells and endothelial cells are prominent.

Although sections from the spleen showed some autolysis, expansion of the red pulp and atrophy of the white pulp was noted. Germinal centers were not observed. Abundant pigment-containing macrophages and some haemophaghocytosis was evident in the red pulp and parasitized red cells were plentiful (Figure 5a). There was no necrosis or fibrin deposition in the spleen.

There were many PRBC's in the liver sinusoids with haemozoin pigment in Kupffer cells and evidence of haemophagocytosis. The portal tracts and sinusoids had moderate chronic lymphoplasmacytic inflammation. Overall the liver was non-cirrhotic but with severe macrovesicular steatosis. No cholestasis, regional necrosis or thrombotic microangiopathy was observed (Figure 5b).

The renal cortex showed dilated and congested blood vessels. Many PRBC were observed within glomerular capillaries with pigment deposition in the mesangium. There was no evidence of thrombotic microangiopathy (disseminated intravascular coagulation, DIC). The tubules showed acute tubular necrosis and regeneration. There were a small number of eosinophilic intra-tubular casts (Figure 5c).

Sequestration of PRBC's was evident in the small vessels of the heart (Figure 5d). Endothelial cells were prominent as sometimes observed in patients with sepsis whose cells are responding to generalized stimuli [18]. There was no evidence of myocarditis and the heart muscle fibers appeared normal. There was focal petechial haemorrhage in the subendocardium, which may relate to resuscitation, or be secondary to malaria.

The adrenal gland appeared active with eosinophilic cytoplasm in the fasciculata layer with no evidence of PRBC sequestration or of parenchymal haemorrhage. Samples of lung, intestine or bone marrow were not available for histopathology examination.

The overall picture was one of systemic malaria infection with multi-organ damage, particularly in the brain where there was much vascular rupture and petechial haemorrhaging.

## Conclusions

Fatal human knowlesi malaria has hitherto not been reported at post-mortem, so this report may increase our understanding of severe and fatal malaria whatever species cause these syndromes. Interestingly, vivax malaria is also becoming recognized as causing severe and sometimes fatal infection in a significant proportion of individuals, suggesting re-evaluation of the dogma that falciparum infection may be the only important cause of fatal disease [19-21].

The WHO classification for severe falciparum malaria would have included this individual because he had several qualifying features including hyperparasitaemia (~10% infected erythrocytes), renal impairment, jaundice and ARDS, although coma was not prominent in the history [3,4,22]. Commonly, dengue haemorrhagic fever can cause some of these signs, but was excluded by investigations. Co-infection of knowlesi with falciparum was also excluded by specific PCR and immunohistochemical examination.

The beefy appearance of lung tissue in this case is consistent with respiratory distress syndrome, but could not be confirmed histologically. Changes in the kidney of some recovering areas of acute tubular injury are consistent with the observation of renal impairment reported in other severe cases of knowlesi malaria[3] and an underlying process of acute tubular necrosis related to systemic shock. There was disorganized white pulp in spleen perhaps as part of a wider stress response [18].

Despite the absence of antecedent established coma, cerebral pathology in this case is very similar to that observed in fatal falciparum infections, confirming that it was important to exclude this co-infection. Although there was obvious accumulation/sequestration of infected erythrocytes in capillaries and venules, there were also some differences between these appearances and those seen with fatal falciparum infections. For example, there was no platelet clumping, no notable thrombi, and uninfected erythrocytes were also interspersed with infected cells. Neither was up-regulation of ICAM-1 expression detected in endothelial cells, suggesting that the mechanism of sequestration/accumulation of infected erythrocytes in knowlesi infections needs further investigation. In falciparum cerebral malaria, display of ICAM-1 by up regulated endothelial cells mediates adhesion to parasitized red cells [19].

Hyperparasitaemia is a marker of severe falciparum malaria. It is also apparent in severe knowlesi infections [3], but is less commonly observed with severe vivax malaria. The 24 hr replicative cycle of asexual knowlesi may contribute to the rapidity with which hyperparasitaemia and clinical complications ensue. This shorter cycle compared with falciparum and vivax infections may overcome the reduced multiplicative capacity associated with the fewer merozoites generated by *P. knowlesi *(up to 16 per mature schizont) compared with *P. falciparum *(up to 36). Although up to 24 merozoites per schizont have been observed in *Plasmodium vivax*, parasitaemia is restricted to reticulocytes by limitation of invasion pathways in this species, rather than merozoite number.

Hyperparasitaemia in falciparum malaria is often explained by protection of late stage parasites from the filtering action of the spleen. Unlike *P. falciparum *infections, all asexual developmental stages are seen in the peripheral circulation of knowlesi-infected patients [10]. Hyperparasitaemia in knowlesi malaria may not be modulated by splenic clearance in a similar manner as suggested for other non-sequestering human infections.

As with many fatal cases of falciparum malaria, malaria pigment was evident in blood films and was present in circulating leucocytes (~40%), tissue sections and organ specific macrophages. Reticuloendothelial changes in both liver and spleen were associated with pigment, accumulation of red cells and laden macrophages, and some inflammation in liver portal tracts was observed. Circulating pigment has been variably implicated as an indicator of poor prognosis for falciparum malaria [23-25] and there is evidence to support pigment-induced immuno-suppression, particularly of pigment-laden macrophages and monocytes [26-28]. It may be that the associations between parasitaemia, pigmentaemia and disease severity are more quantifiable in knowlesi compared with falciparum infections, where pigmented parasites are sequestered from peripheral blood samples and, therefore, unreliably quantifiable. In vivax malaria, pigment in parasites often appears more dispersed and it is rarely reported as a correlate of disease [29].

Among the malaria parasites of humans, cytoadherence is purportedly unique to *P. falciparum*, resulting in sequestration of all but early trophozoite stages in falciparum malaria. Cytoadherence is often implicated in malaria pathology and is mediated by the expression of variable *var *gene products (of the PfEMP-1 family) at the surface of infected host red blood cells with concomitant expression of post-capillary endothelial cell receptors [15,30-32]. Although not easily quantifiable, partial sequestration was observed in this fatal case of knowlesi malaria as evidenced by an abundance of large pigment bearing parasites on the blood film obtained post-mortem and as accumulations in the microvasculature. We hypothesize that partial sequestration may be due to *P. knowlesi *infected cell agglutination mediated by variant surface antigens of *P. knowlesi *SICA *var *genes (orthologues of the PfEMP-1 family) [33,34]. Importantly, in this fatal case of *P. knowlesi*, up-regulation of ICAM-1 was not detected, nor was there evidence for cytoadherence as parasitized cells were not marginalized and were interspersed with uninfected erythrocytes in smaller vessels.

Cerebral malaria and severe acute anaemia are often peculiar to falciparum infection while organ, respiratory and metabolic dysfunction are common with severe knowlesi, vivax and other forms of severe falciparum infection [3,4,19,35-40]. Although late stages of parasites may be visible in the blood in falciparum infections, they are rare and associated with a poor prognosis. Testing the relative contribution of virulence factors to the development of severe malaria including, cytoadherence, hyperparasitaemia, cerebral malaria, organ failure, metabolic and respiratory dysfunction and anaemia was previously difficult without comparative information from severe malaria caused by another species [16,41-44]. Severe and fatal cases of knowlesi malaria will add much needed perspective to what is known of malaria pathophysiology.

The need to develop knowlesi-specific diagnosis, treatment and management guidelines is urgent. A recent prospective clinical study in Sarawak Malaysian Borneo revealed that approximately 1:10 patients infected with *P. knowlesi *present with or develop severe symptoms and 1-2% of cases are fatal [4]. Complications in survivors included ARDS, liver or renal dysfunction, hypotension with or without parasitaemia >100,000/uL. Complications in all fatal knowlesi cases had either clinical or laboratory evidence of abdominal pain, combined hepatorenal dysfunction and hyperparasitaemia [3,4]. There is a prospect that *P. knowlesi *may emerge as a human pathogen beyond its current zoonotic manifestations [11,12], specific surveillance, control and clinical guidelines are necessary to contain potential larger outbreaks.

## Consent

Approval to use this case for educational purposes was obtained from the Deputy Director for Health, Sabah State Department of Health, Ministry of Health, Malaysia.

## Competing interests

The authors declare that they have no competing interests.

## Authors' contributions

JCS collected and collated case information and details and obtained consent. JH performed the post mortem, provided gross pathology images and provided the autopsy report. PCD, ZSR, PC and AP performed PCR, Immunohistochemistry and sample processing. SK, SBL, ANZ and WKT prepared and reviewed the clinical, histopathology and pathology reports. SBL and BS provided descriptions and information for the manuscript. JCS and SK wrote the manuscript. All authors have read and approved the final manuscript.

## References

[B1] Snow RW Guerra CA Mutheu JJ Hay SI International funding for malaria control in relation to populations at risk of stable *Plasmodium falciparum *transmission Plos Med 2008 5 e142 10.1371/journal.pmed.0050142 18651785 PMC2488181

[B2] World Health Organization, World Malaria Report 2008

[B3] Cox-Singh J Davis TM Lee KS Shamsul SS Matusop A Ratnam S Rahman HA Conway DJ Singh B *Plasmodium knowlesi *malaria in humans is widely distributed and potentially life threatening Clin Infect Dis 2008 46 165 171 10.1086/524888 18171245 PMC2533694

[B4] Daneshvar C Davis C Cox-Singh J Rafa'ee M Zakaria S Divis P Singh B Clinical and laboratory features of human *Plasmodium falciparum *infections Clin Infect Dis 2009 49 852 860 10.1086/605439 19635025 PMC2843824

[B5] Coatney GR Collins WE Warren M Contacos PG The primate malarias 1971 Bethesda, MD: U.S. Department of Health, Education and Welfare, National Institutes of Health

[B6] Langhorne J Cohen S *Plasmodium knowlesi *in the marmoset (*Callithrix jacchus*) Parasitology 1979 78 67 76 10.1017/S0031182000048599 33359

[B7] Ozwara H Langermans JA Maamun J Farah IO Yole DS Mwenda JM Weiler H Thomas AW Experimental infection of the olive baboon (*Papio anubis*) with *Plasmodium knowlesi*: severe disease accompanied by cerebral involvement Am J Trop Med Hyg 2003 69 188 194 13677374

[B8] Miller LH Fremount HN Luse SA Deep vascular schizogony of *Plasmodium knowlesi *in *Macaca mulatta*. Distribution in organs and ultrastructure of parasitized red cells Am J Trop Med Hyg 1971 20 816 824 5002246 10.4269/ajtmh.1971.20.816

[B9] Singh B Kim Sung L Matusop A Radhakrishnan A Shamsul SS Cox-Singh J Thomas A Conway DJ A large focus of naturally acquired *Plasmodium knowlesi *infections in human beings Lancet 2004 363 9414 1017 1024 10.1016/S0140-6736(04)15836-4 15051281

[B10] Lee KS Cox-Singh J Singh B Morphological features and differential counts of *Plasmodium knowlesi *parasites in naturally acquired human infections Malar J 2009 8 73 10.1186/1475-2875-8-73 19383118 PMC2676309

[B11] Cox-Singh J Singh B Knowlesi malaria: newly emergent and of public health importance? Trends Parasitol 2008 24 406 410 10.1016/j.pt.2008.06.001 18678527 PMC2843823

[B12] McCutchan FE Is monkey malaria from Borneo an emerging human disease? Future Microbiol 2008 3 115 118 10.2217/17460913.3.2.115 18366329

[B13] Newton CR Krishna S Severe falciparum malaria in children: current understanding of pathophysiology and supportive treatment Pharmacol Ther 1998 79 1 53 10.1016/S0163-7258(98)00008-4 9719344

[B14] Taylor TE Fu WJ Carr RA Whitten RO Mueller JS Fosiko NG Lewallen S Liomba NG Molyneux ME Differentiating the pathologies of cerebral malaria by postmortem parasite counts Nat Med 2004 10 143 145 10.1038/nm986 14745442

[B15] Turner GD Morrison H Jones M Davis TM Looareesuwan S Buley ID Gatter KC Newbold CI Pukritayakamee S Nagachinta B White NJ Berendt AR An immunohistochemical study of the pathology of fatal malaria. Evidence for widespread endothelial activation and a potential role for intercellular adhesion molecule-1 in cerebral sequestration Am J Pathol 1994 145 1057 1069 7526692 PMC1887431

[B16] White N Sherman IW Malaria pathophysiology Malaria: Parasite Biology, Pathogenesis and Protection 1998 Washington DC: ASM Press 371 385

[B17] Genrich GL Guarner J Paddock CD Shieh WJ Greer PW Barnwell JW Zaki SR Fatal malaria infection in travelers: novel immunohistochemical assays for the detection of *Plasmodium falciparum *in tissues and implications for pathogenesis Am J Trop Med Hyg 2007 76 251 259 17297032

[B18] Lucas S The autopsy pathology of sepsis-related death Current Diagnostic Pathology 2007 13 375 388 10.1016/j.cdip.2007.06.001

[B19] Anstey NM Handojo T Pain MC Kenangalem E Tjitra E Price RN Maguire GP Lung injury in vivax malaria: pathophysiological evidence for pulmonary vascular sequestration and posttreatment alveolar-capillary inflammation J Inf Dis 2007 195 589 596 10.1086/510756 PMC2532499 17230420

[B20] Beg MA Khan R Baig SM Gulzar Z Hussain R Smego RA Jr Cerebral involvement in benign tertian malaria Am J Trop Med Hyg 2002 67 3 230 232 12408660 10.4269/ajtmh.2002.67.230

[B21] Genton B D'Acremont V Rare L Baea K Reeder JC Alpers MP Muller I *Plasmodium vivax *and mixed infections are associated with severe malaria in children: a prospective cohort study from Papua New Guinea Plos Med 2008 5 e127 10.1371/journal.pmed.0050127 18563961 PMC2429951

[B22] WHO Severe falciparum malaria. World Health Organizsation, Communicable Diseases Cluster Trans R Soc Trop Med and Hyg 2000 94 Suppl 1 S1 90 11103309

[B23] Kremsner PG Valim C Missinou MA Olola C Krishna S Issifou S Kombila M Bwanaisa L Mithwani S Newton CR Agbenyega T Pinder M Bojang K Wypij D Taylor T Prognostic value of circulating pigmented cells in african African children with malaria J Inf Dis 2009 199 142 150 10.1086/595295 PMC3755538 19086817

[B24] Lyke KE Diallo DA Dicko A Kone A Coulibaly D Guindo A Cissoko Y Sangare L Coulibaly S Dakouo B Taylor T Doumbo OK Plowe C Association of intraleukocytic *Plasmodium *falciparum malaria pigment with disease severity, clinical manifestations, and prognosis in severe malaria Am J Trop Med Hyg 2003 69 253 259 14628940

[B25] Nguyen PH Day N Pram TD Ferguson DJ White NJ Intraleucocytic malaria pigment and prognosis in severe malaria Trans R Soc Trop Med Hyg 1995 89 200 204 10.1016/0035-9203(95)90496-4 7778149

[B26] Awandare GA Ouma Y Ouma C Were T Otieno R Keller CC Davenport GC Hittner JB Vulule J Ferrell R Ong'echa JM Perkins D Role of monocyte-acquired hemozoin in suppression of macrophage migration inhibitory factor in children with severe malarial anemia Infect Immun 2007 75 201 210 10.1128/IAI.01327-06 17060471 PMC1828375

[B27] Carney CK Schrimpe AC Halfpenny K Harry RS Miller CM Broncel M Sewell SL Schaff JE Deol R Carter MD Wright DW The basis of the immunomodulatory activity of malaria pigment (hemozoin) J Biol Inorg Chem 2006 11 917 929 10.1007/s00775-006-0147-0 16868743

[B28] Deshpande P Shastry P Modulation of cytokine profiles by malaria pigment--hemozoin: role of IL-10 in suppression of proliferative responses of mitogen stimulated human PBMC Cytokine 2004 28 205 213 10.1016/j.cyto.2004.08.002 15566949

[B29] McGready R Davison BB Stepniewska K Cho T Shee H Brockman A Udomsangpetch R Looareesuwan S White NJ Meshnick SR Nosten F The effects of *Plasmodium falciparum *and *P. vivax *infections on placental histopathology in an area of low malaria transmission Am J Trop Med Hyg 2004 70 398 407 15100454

[B30] Su XZ Heatwole VM Wertheimer SP Guinet F Herrfeldt JA Peterson DS Ravetch JA Wellems TE The large diverse gene family var encodes proteins involved in cytoadherence and antigenic variation of *Plasmodium falciparum*-infected erythrocytes Cell 1995 82 89 100 10.1016/0092-8674(95)90055-1 7606788

[B31] Flick K Chen Q var genes, PfEMP1 and the human host Mol Biochem Parasitol 2004 134 3 9 10.1016/j.molbiopara.2003.09.010 14747137

[B32] Ockenhouse CF Tegoshi T Maeno Y Benjamin C Ho M Kan KE Thway Y Win K Aikawa M Lobb RR Human vascular endothelial cell adhesion receptors for *Plasmodium falciparum*-infected erythrocytes: roles for endothelial leukocyte adhesion molecule 1 and vascular cell adhesion molecule 1 J Exp Med 1992 176 1183 1189 10.1084/jem.176.4.1183 1383378 PMC2119387

[B33] Pain A Bohme U Berry AE Mungall K Finn RD Jackson AP Mourier T Mistry J Pasini EM Aslett MA Balasubrammaniam S Borgwardt K Brooks K Carret C Carver TJ Cherevach I Chillingworth T Clark TG Galinski MR Hall N Harper D Harris D Hauser H Ivens A Janssen CS Keane T Larke N Lapp S Marti M Moule S Meyer IM Ormond D Peters N Sanders M Sanders S Sargeant TJ Simmonds M Smith F Squares R Thurston S Tivey AR Walker D White B Zuiderwijk E Churcher C Quail MA Cowman AF Turner CMR Rajandream MA Kocken CHM Thomas AW Newbold CI Barrell BG Berriman M The genome of the simian and human malaria parasite *Plasmodium knowlesi* Nature 2008 455 799 803 10.1038/nature07306 18843368 PMC2656934

[B34] Barnwell JW Howard RJ Miller LH Altered expression of *Plasmodium knowlesi *variant antigen on the erythrocyte membrane in splenectomized rhesus monkeys J Immunol 1982 128 224 226 6172478

[B35] Price L Planche T Rayner C Krishna S Acute respiratory distress syndrome in *Plasmodium vivax *malaria: case report and review of the literature Trans R Soc Trop Med Hyg 2007 101 655 659 10.1016/j.trstmh.2007.02.014 17433389

[B36] Coltel N Combes V Hunt NH Grau GE Cerebral malaria -- a neurovascular pathology with many riddles still to be solved Curr Neurovasc Res 2004 1 91 110 10.2174/1567202043480116 16185187

[B37] Deitsch KW Hviid L Variant surface antigens, virulence genes and the pathogenesis of malaria Trends Parasitol 2004 20 562 566 10.1016/j.pt.2004.09.002 15522665

[B38] Idro R Jenkins NE Newton CR Pathogenesis, clinical features, and neurological outcome of cerebral malaria Lancet Neurol 2005 4 827 840 10.1016/S1474-4422(05)70247-7 16297841

[B39] Sun G Chang WL Li J Berney SM Kimpel D Heyde HC van der Inhibition of platelet adherence to brain microvasculature protects against severe *Plasmodium berghei *malaria Infect Immun 2003 71 6553 6561 10.1128/IAI.71.11.6553-6561.2003 14573677 PMC219602

[B40] Taoufiq Z Gay F Balvanyos J Ciceron L Tefit M Lechat P Mazier D Rho kinase inhibition in severe malaria: thwarting parasite-induced collateral damage to endothelia J Inf Dis 2008 197 1062 1073 10.1086/528988 18419473

[B41] Beeson JG Brown GV Pathogenesis of *Plasmodium falciparum *malaria: the roles of parasite adhesion and antigenic variation Cell Mol Life Sci 2002 59 258 271 10.1007/s00018-002-8421-y 11915943 PMC11146193

[B42] Jensen AT Magistrado P Sharp S Joergensen L Lavstsen T Chiucchiuini A Salanti A Vestergaard LS Lusingu JP Hermsen R Sauerwein R Chritensen J Nielsen MA Hviid l Sutherland C Staalsoe T Theander TG *Plasmodium falciparum *associated with severe childhood malaria preferentially expresses PfEMP1 encoded by group A var genes J Exp Med 2004 199 1179 1190 10.1084/jem.20040274 15123742 PMC2211911

[B43] Newton CR Taylor TE Whitten RO Pathophysiology of fatal falciparum malaria in African children Am J Trop Med Hyg 1998 58 673 683 9598460 10.4269/ajtmh.1998.58.673

[B44] Clark IA Alleva LM Is human malarial coma caused, or merely deepened, by sequestration? Trends Parasitol 2009 25 314 318 10.1016/j.pt.2009.04.003 19541540

